# Comparative analysis of laparoscopic and open enucleation for pediatric solid pseudopapillary neoplasm: a retrospective study

**DOI:** 10.3389/fped.2025.1695810

**Published:** 2025-11-05

**Authors:** Xiaogang Zhou, Yi Sun, Jianlei Chen, Peng Cai, Haowei Zhao, Yuliang Jiang, Qi Wang, Menglei Zhu, Jie Zhu, Zhenwei Zhu

**Affiliations:** ^1^ Department of General Surgery, Children’s Hospital of Soochow University, Suzhou, Jiangsu, China; ^2^ Department of Urology, Children’s Hospital of Soochow University, Suzhou, Jiangsu, China

**Keywords:** pediatric, solid pseudopapillary neoplasm, enucleation, pancreatic fistula, laparoscopic

## Abstract

**Objective:**

This study compares the efficacy and safety of laparoscopic enucleation (LEN) vs. open enucleation (OEN) for pediatric solid pseudopapillary neoplasm (SPN) of the pancreas, aiming to provide clinical evidence for optimizing treatment strategies.

**Methods:**

A retrospective analysis evaluated clinical data from 20 pediatric SPN patients undergoing enucleation at the Children's Hospital of Soochow University, with 9 in the LEN group and 11 in the OEN group. Data included baseline characteristics, intraoperative parameters, postoperative outcomes, and complications.

**Results:**

Baseline characteristics were comparable between groups (*p* > 0.05), with a median age of 11 years, and 75.0% female patients. The LEN group exhibited significantly reduced intraoperative blood loss (50.00 mL vs. 90.00 mL, *p* = 0.029) and postoperative pain duration (3.00 days vs. 5.00 days, *p* = 0.037) compared to the OEN group. No significant differences were observed in operative time (LEN: 240.00 min vs. OEN: 255.00 min, *p* = 0.790), hospital stay (LEN: 14.00 days vs. OEN: 15.00 days, *p* = 0.620), or pancreatic fistula incidence (LEN: 22.2% vs. OEN: 18.2%, *p* = 1.000). No grade C pancreatic fistulae, tumor recurrence, or pancreatic dysfunction occurred in either group.

**Conclusion:**

LEN reduces blood loss and postoperative pain in pediatric SPN treatment with comparable safety to OEN. Larger-scale studies with extended follow-up durations are needed to confirm its long-term efficacy.

## 1 Introduction

SPN is a rare pancreatic tumor (1), predominantly affecting young female patients and rarely occurring in children (2). It is characterized by low malignant potential and a favorable prognosis (3). Epidemiological data indicate that SPN accounts for 0.9%–2.7% of pancreatic tumors, with a low global incidence and a female-to-male ratio of approximately 9.8:1 (4). Advances in imaging technologies and increased clinical awareness have significantly improved the diagnostic rate of SPN in recent years (5, 6). In children, SPN presents with varied clinical manifestations, most commonly abdominal pain, abdominal mass, or incidental imaging findings, with some patients experiencing gastrointestinal symptoms due to tumor compression of adjacent organs (7, 8). Although SPN generally carries a favorable prognosis, with over 90% of patients achieving long-term survival post-resection (9, 10), the risks of local invasion and distant metastasis warrant attention.

Complete surgical resection is the recommended treatment for pediatric SPN (11, 12). However, traditional surgical approaches may impact postoperative pancreatic secretory function, a critical consideration in pediatric patients with longer life expectancies compared to adults. In 1898, Ernesto Tricomi first reported pancreatic tumor enucleation (13), a procedure associated with minimal trauma and maximal preservation of pancreatic function, widely applied to benign or low-grade malignant pancreatic tumors, including SPN (8, 14). With recent advancements in minimally invasive techniques, LEN has shown potential in adult SPN treatment, offering reduced trauma and intraoperative blood loss (15). However, studies on enucleation in pediatric SPN are scarce (8), and comparative analyses of LEN vs. OEN regarding efficacy and safety are particularly limited. This is attributed to the rarity of pediatric SPN, the fragility of pediatric pancreatic tissue compared to adults, and the smaller anatomical structures, which demand greater surgical precision.

This study represents the first comparative analysis of OEN and LEN in pediatric SPN. Through a retrospective review of clinical data from 20 pediatric SPN patients who underwent enucleation at our institution between January 2015 and May 2024, this study aims to provide clinical evidence to optimize treatment strategies for pediatric SPN.

## 2 Patients and methods

This study retrospectively collected clinical data from pediatric patients who underwent enucleation for SPN at the Children's Hospital of Soochow University between January 2015 and May 2024. Data encompassed demographic information, intraoperative details, postoperative complications, and follow-up outcomes related to pancreatic endocrine and exocrine function as well as SPN recurrence. Surgical indications included: 1) preoperative imaging suggesting a pancreatic mass consistent with SPN; 2) preoperative imaging unable to definitively characterize the tumor, with intraoperative rapid pathology confirming SPN; or 3) preoperative imaging or intraoperative exploration indicating a tumor margin at least 3 mm from the main pancreatic duct. Throughout the study period, no significant changes occurred in the primary operating surgeons. All procedures were performed by experienced pancreatic surgeons. Postoperative specimens were examined by specialized pathologists and confirmed as SPN. All patients were followed up for at least one year with complete clinical data. This study was approved by the Medical Ethics Committee of the Children's Hospital of Soochow University (Ethics No.: 2025CS164).

## 3 Research methods

Both groups had the following data collected: age, sex, BMI, preoperative clinical manifestations, and preoperative imaging characteristics, including tumor maximum diameter measured by CT or MRI, tumor location in the pancreas, and imaging features. Surgical data included operative time, intraoperative blood loss, postoperative somatostatin use duration, drainage duration, postoperative pain duration, and length of hospital stay. Complications were categorized into early postoperative complications, primarily pancreatic fistula [graded B or C per the International Study Group on Pancreatic Surgery (ISGPS) guidelines (16)], wound infection, early postoperative bowel obstruction, intra-abdominal infection, and reoperation. Long-term complications included pancreatic endocrine and exocrine function, tumor recurrence, postprandial bloating, abdominal pain, diarrhea, constipation, and decreased appetite.

## 4 Surgical techniques

### 4.1 Laparoscopic surgery group

**Figure 1:**
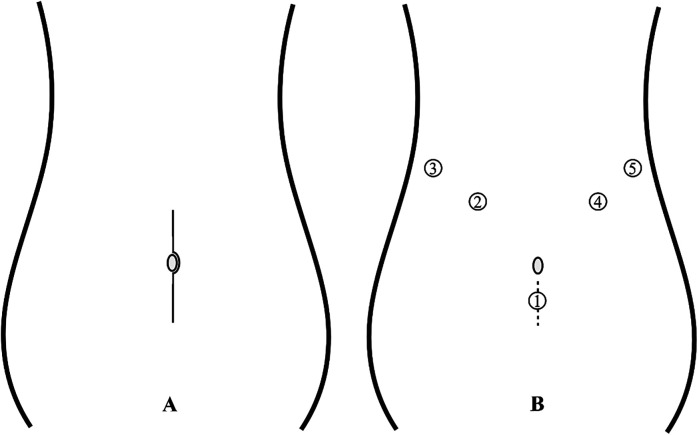
**(A)** Open surgical incision: a length of 12–15 cm. **(B)** Trocar placement for laparoscopic surgery: 1: Endoscope; 2: Operating forceps; 3-5: Assistant port; The dashed line in the figure denotes the extended incision made for tumor extraction, approximately 3–5 cm in length.

**Figure 2:**
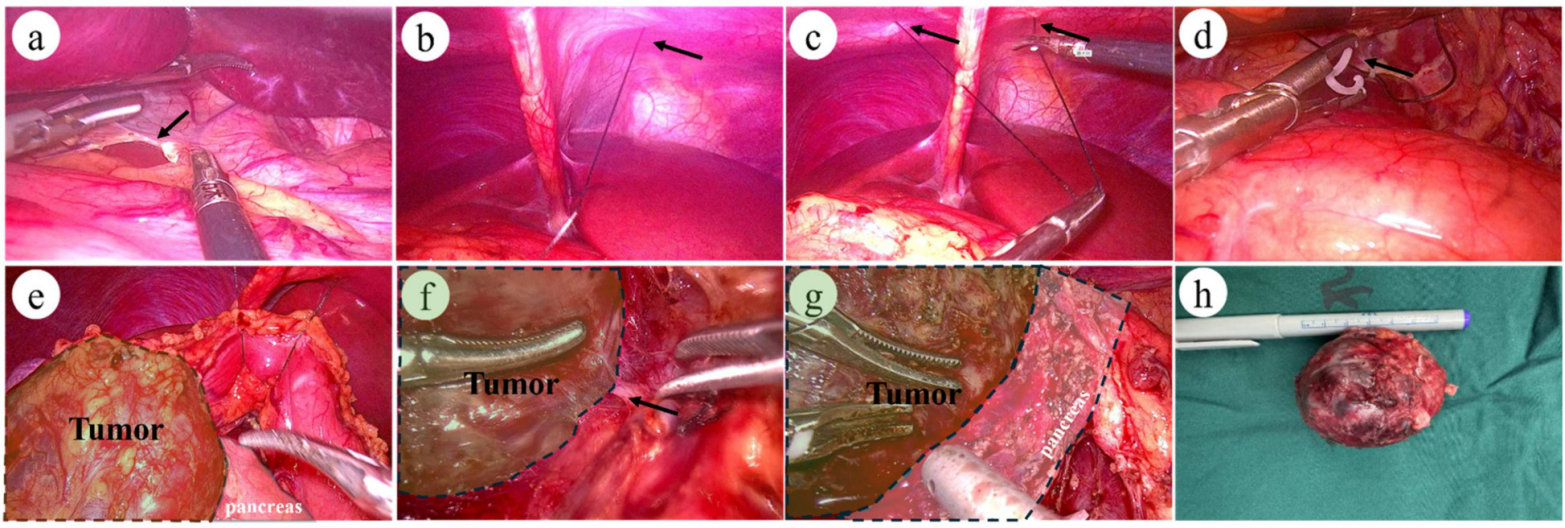
Intraoperative images of laparoscopic surgery: **(a)** arrow indicates: division of the hepatogastric ligament. **(b–c)** Arrows indicate: Puncture and placement of a 2-0 suture with needle at the subxiphoid abdominal wall. **(d)** Fixation of the suture midpoint to the hepatogastric ligament posterior to the stomach. **(e)** Complete exposure of the pancreas and pancreatic tumor. **(f)** Arrow indicates: Clear laparoscopic visualization of the tumor's feeding vessels. (**g)** Demonstration of the interface between the tumor and normal pancreatic tissue. **(h)** Postoperative tumor specimen: Complete tumor resection.

Patients were placed in the supine position under general anesthesia with standard disinfection. The trocar placement is illustrated in Figure 1B: a 10-mm trocar was inserted 2 cm below the umbilicus, 5-mm trocars were placed below the costal margin on both sides of the anterior axillary line and at the left midclavicular line, and a 10-mm trocar was placed at the right midclavicular line. The gastrocolic ligament was opened to expose the pancreas and locate the tumor. The hepatogastric ligament was accessed, and a 2-0 silk suture was passed through the abdominal wall, secured behind the stomach to the hepatogastric ligament, and tightened to suspend the stomach and liver, enhancing surgical field exposure. An ultrasonic scalpel was used to meticulously dissect the tumor from the pancreatic tissue. Hemostasis was achieved using the ultrasonic scalpel, bipolar coagulation, or suturing for oozing or active bleeding to maintain a clear field. Post-tumor resection, the dissection surface was irrigated with warm saline, dried with gauze, and inspected for pancreatic juice leakage to assess main pancreatic duct integrity, with intraoperative repair performed if needed. Silicone drainage tubes were placed under the liver and at the pancreatic dissection site. Depending on tumor size, the infraumbilical incision was enlarged to 3–5 cm for specimen retrieval (Figure 2). Intraoperative rapid pathology was routinely performed; if malignancy was confirmed, extended resection was conducted with a modified surgical approach.

### 4.2 Open surgery group

Patients were placed in the supine position under general anesthesia with standard disinfection. A 12–15 cm midline upper abdominal incision was made to enter the peritoneal cavity layer by layer (Figure 1A). Abdominal retractors were used to expose the surgical field, with subsequent steps mirroring those of the laparoscopic approach.

## 5 Statistical analysis

Statistical analysis was performed using R 4.3.0 software. Given the small sample size, continuous variables were expressed as medians (interquartile range, *Q*₁–*Q*₃) and analyzed using the Mann–Whitney *U* test to ensure robust results. Categorical variables were reported as frequencies and percentages (*n*, %) and compared using Fisher's exact test based on sample size and distribution. A two-sided *p*-value < 0.05 was considered statistically significant.

## 6 Results

### 6.1 General information

This study included 20 pediatric patients with SPN, with 9 undergoing LEN and 11 undergoing traditional OEN. Baseline characteristics revealed a median age of 11 years, a female predominance of 75.0%, and a median BMI of 18.00 kg/m^2^. Preoperative clinical manifestations were primarily abdominal pain (17/20, 85.0%), followed by vomiting (9/20, 45.0%), with bloating, jaundice, and abdominal mass being less common (2/20, 10.0%). Imaging findings showed a median tumor diameter of 4.10 cm, with tumors most frequently located in the pancreatic head (9/20, 45.0%), followed by the tail (5/20, 25.0%), body (4/20, 20.0%), and neck (2/20, 10.0%). Radiologically, solid tumors predominated (12/20, 60.0%). No significant differences were observed between the OEN and LEN groups for these parameters (Table 1).

**Table 1:** Baseline characteristics of the study population.

Variables	Total (*n* = 20)	OEN (*n* = 11)	LEN (*n* = 9)	*p*
Age, years, *M* (*Q*₁, *Q*₃)	11.00 (9.00, 12.00)	12.00 (9.50, 12.50)	11.00 (9.00, 12.00)	0.615
BMI, kg/m^2^, *M* (*Q*₁, *Q*₃)	18.00 (16.48, 20.72)	19.80 (16.90, 21.00)	17.30 (16.60, 20.70)	0.494
Gender, *n* (%)				1.000
Female	15 (75.0)	8 (72.7)	7 (77.8)	
Male	5 (25.0)	3 (27.3)	2 (22.2)	
Clinical manifestations				
Abdominal pain, *n* (%)				0.218
No	3 (15.0)	3 (27.3)	0 (0.0)	
Yes	17 (85.0)	8 (72.7)	9 (100.0)	
Abdominal distension, *n* (%)				0.479
No	18 (90.0)	9 (81.8)	9 (100.0)	
Yes	2 (10.0)	2 (18.2)	0 (0.0)	
Vomiting, *n* (%)				0.653
No	11 (55.0)	7 (63.6)	4 (44.4)	
Yes	9 (45.0)	4 (36.4)	5 (55.6)	
Jaundice, *n* (%)				1.000
No	18 (90.0)	10 (90.9)	8 (88.9)	
Yes	2 (10.0)	1 (9.1)	1 (11.1)	
Abdominal mass, *n* (%)				0.479
No	18 (90.0)	9 (81.8)	9 (100.0)	
Yes	2 (10.0)	2 (18.2)	0 (0.0)	
Radiological characteristics				
Tumor diameter, cm, *M* (*Q*₁, *Q*₃)	4.10 (3.58, 5.50)	4.10 (3.75, 5.75)	4.10 (3.60, 5.20)	0.594
Tumor location, *n* (%)				0.730
Head	9 (45.0)	4 (36.4)	5 (55.6)	
Neck	2 (10.0)	1 (9.1)	1 (11.1)	
Body	4 (20.0)	2 (18.1)	2 (22.2)	
Tail	5 (25.0)	4 (36.4)	1 (11.1)	
Imaging features, *n* (%)				0.835
Solid	12 (60.0)	7 (63.6)	5 (55.6)	
Cystic	3 (15.0)	2 (18.2)	1 (11.1)	
Cystic-solid	5 (25.0)	2 (18.2)	3 (33.3)	

LEN, laparoscopic enucleation; OEN, open enucleation, *M*, Median; *Q*₁, 1st Quartile; *Q*₃, 3st Quartile.

### 6.2 Intraoperative and postoperative outcomes

The overall median operative time was 252.50 min, slightly shorter in the LEN group (240.00 min) than in the OEN group (255.00 min, *p* = 0.790), though not statistically significant. The overall median intraoperative blood loss was 50.00 mL, significantly lower in the LEN group (50.00 mL) compared to the OEN group (90.00 mL, *p* = 0.029). The overall median postoperative hospital stay was 14.00 days, shorter in the LEN group (14.00 days) than in the OEN group (15.00 days), but the difference was not significant (*p* = 0.620). The median drainage tube removal time was 10 days, with no significant difference between groups. The median postoperative pain duration was 4 days, significantly shorter in the LEN group (3.00 days) compared to the OEN group (5.00 days, *p* = 0.037) (Table 2).

**Table 2:** Operation details and postoperative complications.

Variables	Total (*n* = 20)	OEN (*n* = 11)	LEN (*n* = 9)	*p*
Operative time, min, *M* (*Q*₁, *Q*₃)	252.50 (229.25, 300.00)	255.00 (210.00, 272.50)	240.00 (234.00, 330.00)	0.790
Blood loss, mL, *M* (*Q*₁, *Q*₃)	50.00 (28.75, 92.50)	90.00 (40.00, 110.00)	50.00 (20.00, 50.00)	0.029
Somatostatin duration, days, *M* (*Q*₁, *Q*₃)	9.50 (6.00, 13.00)	12.00 (7.00, 13.50)	8.00 (2.00, 12.00)	0.146
Drainage duration, days, *M* (*Q*₁, *Q*₃)	10.00 (6.75, 15.00)	10.00 (6.50, 14.50)	10.00 (7.00, 15.00)	1.000
Postoperative pain duration, days, *M* (*Q*₁, *Q*₃)	4.00 (3.00, 5.75)	5.00 (4.00, 8.00)	3.00 (2.00, 3.00)	0.037
Postoperative hospital stay, days, *M* (*Q*₁, *Q*₃)	14.00 (12.00, 19.25)	15.00 (13.00, 21.50)	14.00 (12.00, 15.00)	0.620
Early postoperative complications				
Pancreatic fistula, *n* (%)				1.000
B	4 (20.0)	2 (18.2)	2 (22.2)	
C	0 (0.0)	0 (0.0)	0 (0.0)	
Wound infection, *n* (%)	1 (5.0)	1 (9.1)	0 (0.0)	1.000
Intestinal obstruction, *n* (%)	0 (0.0)	0 (0.0)	0 (0.0)	–
Intra-abdominal infection, *n* (%)	0 (0.0)	0 (0.0)	0 (0.0)	–
Reoperation, *n* (%)	0 (0.0)	0 (0.0)	0 (0.0)	–
Long-term complications				
Endocrine dysfunction, *n* (%)	0 (0.0)	0 (0.0)	0 (0.0)	–
Exocrine dysfunction, *n* (%)	0 (0.0)	0 (0.0)	0 (0.0)	–
Tumor recurrence, *n* (%)	0 (0.0)	0 (0.0)	0 (0.0)	–
Postprandial abdominal distension, *n* (%)	1 (5.0)	1 (9.1)	0 (0.0)	1.000
Abdominal pain, *n* (%)	1 (5.0)	1 (9.1)	0 (0.0)	1.000
Diarrhea, *n* (%)	0 (0.0)	0 (0.0)	0 (0.0)	–

LEN, laparoscopic enucleation; OEN, open enucleation; *M*, Median; *Q*₁, 1st Quartile; *Q*₃, 3st Quartile.

### 6.3 Postoperative complications

Regarding early postoperative complications, pancreatic fistula occurred in 4 patients (20.0%), with 7 cases in the OEN group (18.2%) and 4 in the LEN group (22.2%), showing no significant difference in distribution (*p* = 1.000). No grade C pancreatic fistulae were observed. All grade B fistulae were effectively managed with prolonged drainage, total parenteral nutrition, and somatostatin therapy. One patient (5.0%) in the OEN group developed a wound infection, which resolved with conservative treatment and dressing changes. No cases of early postoperative bowel obstruction, intra-abdominal infection, or reoperation were reported in either group. For long-term complications, no patients in either group exhibited pancreatic endocrine or exocrine dysfunction or tumor recurrence. In the OEN group, one patient (5.0%) experienced intermittent abdominal pain, attributed to intestinal adhesions from open surgery during regular outpatient follow-up, and another (5.0%) reported mild postprandial bloating without other symptoms, managed with routine outpatient monitoring (Table 2).

## 7 Discussion

This study is the first to compare the efficacy and safety of LEN vs. OEN in the treatment of pediatric SPN, elucidating differences in intraoperative and postoperative outcomes. The LEN group demonstrated significantly reduced intraoperative blood loss (median 50.00 mL vs. 90.00 mL, *p* = 0.029) and postoperative pain duration (median 3.00 days vs. 5.00 days, *p* = 0.037) compared to the OEN group, suggesting that laparoscopic techniques offer potential advantages in minimizing trauma and enhancing postoperative recovery. These findings align with the minimally invasive benefits of laparoscopic surgery reported in adult SPN studies (15). However, pediatric pancreatic tissue is more fragile than in adults, and the smaller anatomical structures demand greater surgical precision, making the application of LEN in pediatric SPN more challenging.

Operation time were comparable between the two groups, but enucleation, in general, offers shorter operative durations compared to traditional pancreatic surgeries (17). The LEN group exhibited significantly less intraoperative blood loss (50.00 mL vs. 90.00 mL, *p* = 0.029). The pancreas, located predominantly in the retroperitoneum, is deeply positioned with a rich blood supply, rendering it prone to bleeding during surgery. Effective intraoperative exposure (Figure 2) benefited from the magnified field of view provided by laparoscopy, enabling clear visualization and ligation of tumor-feeding vessels (Figure 2F). The use of an ultrasonic scalpel and bipolar coagulation under laparoscopy facilitated prompt hemostasis of surface oozing. For bleeding from the tumor itself, bipolar coagulation proved highly effective. For tumors located superficially or farther from the main pancreatic duct, electrocoagulation or the ultrasonic scalpel was used for hemostasis, with ligation or suturing applied to discrete bleeding points. Laparoscopy clearly delineated the boundary between the tumor and normal pancreatic tissue, allowing dissection along the tumor capsule to avoid encroaching on healthy pancreatic tissue (Figure 2G). This approach not only reduced bleeding but also preserved normal pancreatic tissue and the main pancreatic duct.

The advantages of laparoscopic surgery were further evident in the reduced postoperative pain duration (3.00 days vs. 5.00 days, *p* = 0.037). Laparoscopic surgery involves smaller incisions, and incision size is correlated with postoperative pain. The open surgery group had incisions of approximately 12–15 cm, whereas the laparoscopic group's incisions, primarily for tumor retrieval below the umbilicus, measured about 3–5 cm, significantly smaller than those in the open group. Studies indicate that incisions longer than 10 cm significantly increase the risk of moderate-to-severe pain (18). Additionally, longer incisions are a risk factor for wound infection (19). In our open surgery group, one case (5.0%) developed a wound infection, which resolved with regular dressing changes. Minimally invasive laparoscopic surgery significantly alleviated postoperative pain, enabling earlier ambulation and promoting faster recovery of intestinal function (20).

No significant differences were observed between the LEN and OEN groups in operative time (LEN: 240.00 min vs. OEN: 255.00 min, *p* = 0.790), postoperative hospital stay (LEN: 14.00 days vs. OEN: 15.00 days, *p* = 0.620), or pancreatic fistula incidence (LEN: 22.2% vs. OEN: 18.2%, *p* = 1.000), indicating that LEN is feasible for pediatric SPN with comparable safety to OEN, without increasing postoperative complication rates. However, pancreatic fistula, the primary complication, did not show a significantly reduced incidence with different surgical approaches. In this study, pancreatic fistula rates were 22.2% in the LEN group and 18.2% in the OEN group, and no grade C fistulae. All grade B fistulae were effectively managed with prolonged drainage, total parenteral nutrition, and somatostatin therapy, with no patients requiring reoperation. The incidence of pancreatic fistula in our study was relatively high, which is consistent with previous research indicating that enucleation is associated with a relatively high incidence of pancreatic fistula (8, 14, 21). The nature of enucleation surgery inherently predisposes patients to a higher risk of pancreatic fistula due to the extensive dissection required to remove the tumor while preserving pancreatic tissue (22). The incidence of pancreatic fistula is related to the size of the pancreatic resection surface and the method of handling this surface. Previous studies suggest that laparoscopic techniques, by offering magnified visualization and precise dissection, can reduce pancreatic fistula rates (4.5%) (23). In our study, the size of the tumors did not differ significantly between the two surgical groups, which may contribute to the lack of a significant difference in pancreatic fistula rates between the laparoscopic and open techniques. The resection surface was managed uniformly by open drainage in both groups. This approach was chosen to facilitate the drainage of pancreatic exudate and to monitor for the presence of a fistula. While previous studies have suggested that laparoscopic techniques may reduce the incidence of pancreatic fistula (23), our study's small sample size (*n* = 20) may have limited our ability to detect a significant difference between the two groups. The occurrence of postoperative pancreatic fistula significantly impacts the length of hospital stay for patients. Our study found that the incidence of Grade B pancreatic fistula was similar between the two groups, which likely contributed to the similar postoperative hospital stays. Research has shown that an increased incidence of Grade B pancreatic fistula is associated with prolonged hospitalization (24). Therefore, reducing the incidence of postoperative pancreatic fistula is crucial for minimizing hospital stay duration.

Preventing pancreatic fistulae and optimizing their management are crucial for enucleation procedures. This study found comparable overall pancreatic fistula rates between LEN and OEN, with no statistical difference. However, literature indicates that laparoscopic techniques, with their high-definition visualization and facilitation of precise anatomical dissection, can significantly lower pancreatic fistula rates (4.5%) (23). This advantage likely stems from laparoscopy's superior precision in dissection and hemostasis. Protection of the main pancreatic duct is paramount, as tumors <2–3 mm from the duct increase the risk of intraoperative injury (22). For patients with deeply located tumors <2 mm from the main pancreatic duct, preoperative endoscopic retrograde cholangiopancreatography (ERCP) with pancreatic duct stent placement to mark and protect the duct, or intraoperative ultrasound to locate the duct, is recommended (25, 26). During enucleation of tumors close to the main pancreatic duct, meticulous dissection is essential to avoid injury. Furthermore, pancreatic surgery wound management affects pancreatic fistula incidence. Our center employed open drainage, placing drainage tubes around the pancreas and subhepatic region, with postoperative monitoring of drain amylase levels and output to determine removal timing. The median drainage tube removal time was 10 days post-surgery. Among the 20 patients, four developed grade B pancreatic fistulae, all effectively managed with prolonged drainage, total parenteral nutrition, and somatostatin therapy. To address the issue of postoperative pancreatic fistula and its impact on hospital stay, we recommend that thorough preoperative assessment is essential, utilizing advanced imaging modalities such as enhanced computed tomography (CT), magnetic resonance imaging (MRI), and magnetic resonance cholangiopancreatography (MRCP) to evaluate the relationship between the tumor and the main pancreatic duct. Tumors located within 3 mm of the main pancreatic duct are considered a risk factor for postoperative pancreatic fistula, and we have avoided enucleation in such cases. Our center plan to routinely incorporate intraoperative ultrasound in future surgeries to enhance tumor localization and to measure the distance between the tumor and the main pancreatic duct. This technique also helps in assessing the integrity of the main pancreatic duct (2). Additionally, our center plan to place pancreaticobiliary stents during enucleation, as previous studies have shown that stent placement can reduce the risk of injury to the main pancreatic duct and common bile duct. We will maintain our current practice of open drainage to facilitate the drainage of pancreatic exudate and to monitor for pancreatic fistula. Furthermore, early postoperative measures are equally critical, including ensuring adequate drainage, preventing infection, and administering somatostatin analogs to reduce pancreatic juice secretion.

Compared to adults, long-term preservation of pancreatic endocrine and exocrine function is particularly critical in younger pediatric SPN patients (2, 8). In this study, neither the LEN nor OEN group exhibited postoperative pancreatic dysfunction, underscoring the advantage of enucleation in preserving pancreatic tissue. However, pediatric pancreatic tissue is smaller and more fragile, increasing the risk of intraoperative bleeding or pancreatic juice leakage, which elevates pancreatic fistula risk (8). Our study found a longer postoperative hospital stay in pediatric SPN patients (median 14–15 days) compared to adult studies (8–12.3 days) (17), possibly due to our more conservative pancreatic fistula management strategy and differences in pediatric postoperative recovery needs.

Identification and preservation of the main pancreatic duct and the common bile duct during enucleation of SPN are critical steps. Preoperative assessment plays a vital role in planning the surgical approach. We routinely use MRCP and biliary and pancreatic ultrasound to evaluate the relationship between the tumor and the pancreaticobiliary ducts. A tumor margin of less than 3 mm from the main pancreatic duct is considered a risk factor for ductal injury. In cases where the tumor is in close proximity to the main pancreatic duct, our center have recently adopted the practice of preoperative placement of pancreaticobiliary stents via ERCP to facilitate intraoperative identification and protection of these structures. Intraoperatively, maintaining a clear surgical field and meticulous hemostasis are essential. We utilize bipolar electrocoagulation, which conducts current only between the tips of the forceps, minimizing the risk of thermal injury to adjacent tissues, including the pancreatic and biliary ducts. When dissecting around the tumor, we carefully separate the tumor capsule from the main pancreatic duct and the common bile duct, avoiding clamping these structures. The main pancreatic duct and the common bile duct are relatively resilient tissues, and experienced pancreatic surgeons can usually identify them without difficulty. Some studies have reported the use of preoperative stent placement, intraoperative ultrasound, or intraoperative cholangiography with fluorescence imaging to aid in the identification and protection of these ducts (27, 28). In the event of intraoperative injury to the pancreaticobiliary ducts, immediate repair is attempted. If repair is not feasible, the surgical approach is modified, such as performing a partial pancreatectomy with pancreaticojejunostomy. For SPN located in the head of the pancreas, we also emphasize the importance of preserving the blood supply to the duodenum and the common bile duct, including the pancreaticoduodenal arteries (29). We strive to preserve both the anterior and posterior vascular arcades, with a minimum goal of preserving the posterior arcade. In the event of accidental injury, a standard pancreaticoduodenectomy is promptly performed to prevent postoperative ischemic complications of the duodenum and bile duct.

Enucleation offers several advantages, including reduced intraoperative blood loss, no requirement for digestive tract reconstruction, and a lower incidence of long-term pancreatic endocrine and exocrine insufficiency due to minimal resection of pancreatic tissue. However, as highlighted in our study and corroborated by prior research, enucleation is associated with a higher postoperative pancreatic fistula rate, particularly in cases involving larger tumors or those located near the main pancreatic duct (30, 31). Wang et al. (17) compared 31 patients undergoing enucleation for SPN with 70 patients undergoing standard resections, reporting less intraoperative blood loss and lower rates of long-term pancreatic insufficiency in the enucleation group, but a higher incidence of postoperative pancreatic fistula. Similarly, Cho et al. (24) found that pediatric patients undergoing enucleation had a higher rate of clinically relevant (Grade B/C) postoperative pancreatic fistula compared to those undergoing standard resections. Wu et al. (8) further confirmed that pancreatic fistula is a primary complication following enucleation, with significantly higher rates compared to standard resection groups, though long-term pancreatic function was better preserved in the enucleation group. Additionally, Kwon et al. (32) identified the volume of resected pancreatic tissue as a key risk factor for long-term pancreatic insufficiency, supporting the long-term benefits of enucleation. Our study's findings align with these observations, demonstrating a higher early pancreatic fistula rate in the enucleation group but a lower incidence of long-term pancreatic endocrine and exocrine insufficiency compared to standard resections.

This study is the first retrospective analysis directly comparing LEN and OEN in pediatric SPN, filling a research gap and providing preliminary evidence for optimizing clinical treatment strategies. All procedures were performed by experienced pancreatic surgeons, and postoperative pathology confirmed SPN, ensuring diagnostic consistency and data reliability. The use of the internationally recognized ISGPS pancreatic fistula grading standard enhanced the scientific rigor and comparability of results. However, limitations include the small sample size (*n* = 20), which may have restricted the detection of statistical differences, particularly in complication rates and long-term outcomes. Given the low malignant potential of SPN, the follow-up duration in our study represents a limitation. Our future studies will incorporate longer follow-up periods to provide more definitive insights into recurrence rates. Additionally, the inherent biases of a retrospective design may affect result robustness. Future studies should employ multicenter, prospective designs with larger sample sizes to explore optimized pancreatic fistula management strategies and further evaluate the long-term efficacy and functional preservation of laparoscopic techniques in pediatric SPN, providing more comprehensive guidance for clinical practice.

## 8 Conclusion

LEN demonstrates significant advantages in reducing intraoperative blood loss and postoperative pain in the treatment of pediatric SPN, while maintaining safety and efficacy comparable to OEN. This study provides the first clinical evidence supporting minimally invasive treatment for pediatric SPN, suggesting that laparoscopic techniques are a safe and effective therapeutic option. Future multicenter, large-scale studies are needed to further validate its long-term efficacy and pancreatic function preservation, ultimately optimizing treatment strategies for pediatric SPN.

## Data Availability

The original contributions presented in the study are included in the article/Supplementary Material, further inquiries can be directed to the corresponding authors.
